# Lung Ultrasound and Pleural Artifacts: A Pictorial Review

**DOI:** 10.3390/diagnostics14020179

**Published:** 2024-01-13

**Authors:** Ehsan Safai Zadeh, Christian Görg, Helmut Prosch, Daria Kifjak, Christoph Frank Dietrich, Christian B. Laursen, Hajo Findeisen

**Affiliations:** 1 Department of Biomedical Imaging and Image-Guided Therapy, Medical University of Vienna, Vienna General Hospital, 1090 Vienna, Austria; 2 Interdisciplinary Center of Ultrasound Diagnostics, Clinic for Gastroenterology, Endocrinology, Metabolism and Clinical Infectiology, University Hospital Giessen and Marburg, Philipp University of Marburg, Baldingerstraße, 35037 Marburg, Germany; 3 Department of Radiology, Mass Memorial Medical Center and University of Massachusetts Chan Medical School, Worcester, MA 01655, USA; 4 Department of General Internal Medicine (DAIM), Hirslanden Clinics Bern, Beau Site, Salem and Permanence, 3018 Bern, Switzerland; c.f.dietrich@googlemail.com; 5 Department of Respiratory Medicine, Odense University Hospital, 5000 Odense, Denmark; 6 Odense Respiratory Research Unit, Department of Clinical Research, University of Southern Denmark, 5230 Odense, Denmark; 7 Department for Internal Medicine, Red Cross Hospital Bremen, 28199 Bremen, Germany

**Keywords:** lung ultrasound, pleural artifacts, B-line artifacts, comet-tail artifacts, diagnosis, interstitial pattern

## Abstract

Lung ultrasound is a well-established diagnostic approach used in detecting pathological changes near the pleura of the lung. At the acoustic boundary of the lung surface, it is necessary to differentiate between the primary visualization of pleural parenchymal pathologies and the appearance of secondary artifacts when sound waves enter the lung or are reflected at the visceral pleura. The aims of this pictorial essay are to demonstrate the sonographic patterns of various pleural interface artifacts and to illustrate the limitations and pitfalls of the use of ultrasound findings in diagnosing any underlying pathology.

## 1. Introduction

Lung ultrasound (LUS) is an established diagnostic method for the assessment of pathological changes near the pleura of the lung [1,2]. However, acoustic imaging of the lung is limited compared with other radiological imaging [3]. The diagnostic value of LUS largely depends on the amount of air content in the lung tissue, as air reflects sound waves. At the acoustic boundary of the lung surface, it is necessary to differentiate between the primary visualization of pleural parenchymal pathologies and the appearance of secondary artifacts when sound waves enter the lung or are reflected at the visceral pleura. In particular, the interstitial pattern (IP), comprising “B-line artifacts” (BLAs) and “comet-tail artifacts” (CTAs), is frequently mentioned in the literature. However, these artifacts are nonspecific and may be present in a diverse range of pathologies.

This pictorial essay assesses the sonographic patterns of various pleural interface artifacts in different pathologies and illustrates the limitations and pitfalls of using ultrasound findings in the diagnosis of any underlying pathology.

## 2. Basic Principles and Examination Technique

**Figure 1:**
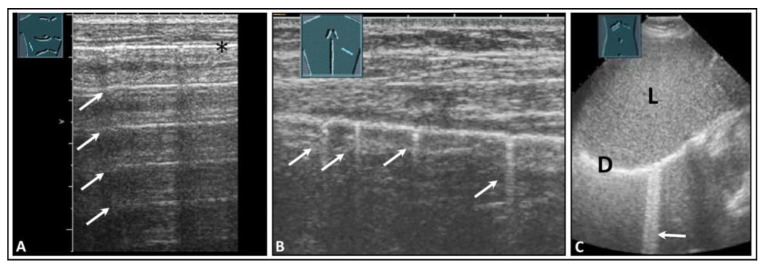
Visualization of pleural artifacts as incidental findings. (**A**) A-lines (arrows), with the top line (*) corresponding to the lung sliding sign. (**B**) Comet-tail artifacts (arrows) originating from the lung sliding sign. (**C**) B-lines (arrow) at the diaphragmatic pleura in the right-sided subcostal acoustic window; L = liver; D = diaphragm.

Healthy lung is characterized by an air content of over 99% [4]. Due to the high impedance difference between the air-filled lung tissue in the normal lung and the tissue of the thoracic wall, there is complete reflection of sound waves, making it impossible to visualize the healthy air-filled lung tissue using ultrasound [5]. At the site of total reflection, an echogenic reflection line can be seen at the lung surface in LUS, and this is referred to as the “air-interface line”. Regular parallel horizontal repetition echoes appear dorsally to the lung surface due to the reverberation phenomenon at the air-interface line and are projected into the lung space, forming the so-called “A-lines”. The distance between the A-lines is constant, but the intensity decreases from the surface downwards (Figure 1A) [6,7].

In addition to the typical physiologic A-lines, isolated vertical artifacts can occur in the form of CTAs and BLAs. These originate from the lung surface. However, it is important to note that the terms “BLA” and “CTA” lack consistent definitions in the literature and are used in varying ways [8,9]. Lichtenstein et al. described these artifacts as long and short types of CTA and clearly stated that a BLA is a long CTA [8,10]. At an international consensus conference in 2012, Volpicelli et al. recommended that the term “CTA” should be avoided for artifacts that originate from the pleural surface and extend to the bottom of the image without fading, moving synchronously with the sliding of the lung, instead favoring “BLA”. Multiple BLAs were defined as a sonographic sign of a so-called “interstitial lung syndrome” [5]. In this paper, however, short CTAs, as defined in the literature, were not considered [5]. Additionally, in terms of terminology, it is inaccurate to refer to an image as a “syndrome”. By definition, a syndrome is a distinguishable combination of symptoms and physical observations that suggest a particular condition, even when the precise underlying cause is not fully understood [11]. To avoid potential misunderstandings, in a World Federation for Ultrasound in Medicine and Biology position paper in 2021 [1], Mathis et al. defined these artifacts more clearly than did Volpicelli et al. According to this position paper, CTAs show a decrease in artifact width from their origin at the air-interface line to the bottom of the image (like a comet) and have a vertical extension of <10 cm (Figure 1B) [1]. CTAs arise from tissue roughness on the ventilated lung surface and are mostly due to scarring [12]. If artifacts run from the air-interface line to the bottom of the image with a consistent width across the entire image and have an extension of >10 cm, they are referred to as B-lines (Figure 1C) [1]. It is considered normal if three or fewer B-lines or CTAs are detected in an ultrasound scan [12].

**Figure 2:**
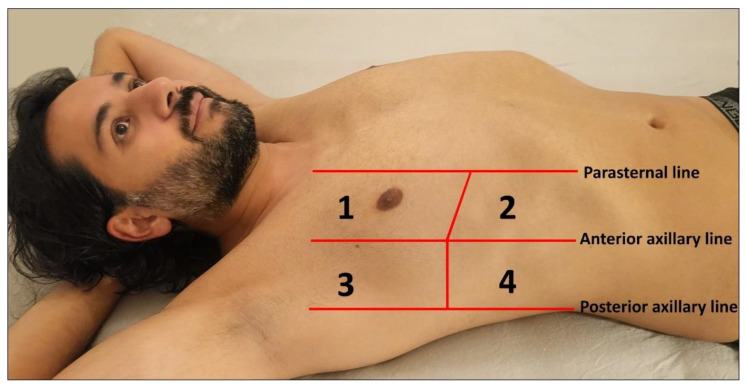
Illustration of an eight-zone lung ultrasound examination. Four chest areas per side are examined to assess the presence of interstitial pattern: Areas 1 and 2 represent the upper anterior and lower anterior chest regions, respectively, while Areas 3 and 4 correspond to the upper lateral and basal lateral chest areas, respectively.

The choice of transducers and different preset or image-optimization software packages for the examination of these artifacts may influence the findings. Sperandeo et al. conducted a study investigating the number of B-lines using both a low- to medium-frequency convex probe (3.5–5.0 MHz) and a high-frequency linear probe (8.0–12.5 MHz) [13]. They found that the number of BLAs was significantly higher when using the convex probes [13]. Essentially, the abdominal probe operating at 3–5 MHz offers a wider field of view of the pleura, while the use of high-frequency transducers (8.0–12.5 MHz) provides superior detection of pleural and subpleural pathological changes [14]. Furthermore, the age of the patients should be considered when setting the examination conditions. In pediatric patients, the small size of the chest makes the use of 7 to 12 MHz linear probes the optimal choice for achieving clear visualization of the pleura and lung surfaces [3,15]. An eight-zone protocol based on international recommendations for point-of-care LUS can be used for the examination (Figure 2) [5].

The appearance of these artifacts changes with a decrease in the lung’s air content. An increase in lung density, caused by, among other disorders, increased cell content or fluid in the interstitium or alveolar space, leads to high-impedance discontinuities in the total reflection at the lung surface [6]. As a result, sound waves are partially reflected in deep zones, producing reverberations on the now-irregular lung surface with minute pleural patches of roughness measuring <5 mm [5,6]. This phenomenon leads to an increasing occurrence of CTAs per ultrasound scan, correlating to the decrease in air content [16]. Lichtenstein et al. defined the presence of more than three B-lines or CTAs as pathological [12]. Furthermore, Volpicelli and colleagues described “interstitial syndrome” as the presence of a minimum of three B-lines in more than two anterior or lateral intercostal spaces on each side of the thorax [5]. However, the number of these artifacts can vary based on several factors, including the ultrasound machine, choice of ultrasound transducer, machine settings, and patient age [14,17,18]. In addition, it is important to determine whether the pattern exists locally or diffusely.

## 3. Underlying Pathology in Artifacts

**Figure 3:**
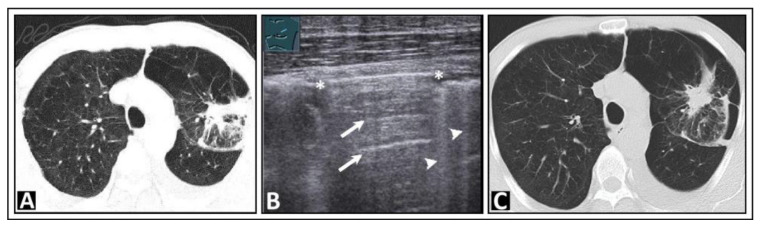
A patient with bronchial carcinoma and localized pneumothorax after lung biopsy. (**A**) Visualization of a lesion of the left lung on chest computed tomography before biopsy. (**B**) A localized pneumothorax with two lung puncture points (*), A-lines dorsal to the free air (arrows), and B-lines behind the lung sliding sign (arrowheads). (**C**) Computed tomography confirms the diagnosis of a pneumothorax.

Based on a systematic review with meta-analysis of 5314 patients, LUS had a sensitivity of 87% and a specificity of 99% for a pneumothorax, compared with chest radiography, which had a sensitivity of 46% and a specificity of 100% [3]. A-lines, in combination with absent lung sliding, can also be observed in the presence of free air in the pleural space, indicative of a pneumothorax (Figure 3) [19].

**Figure 4:**
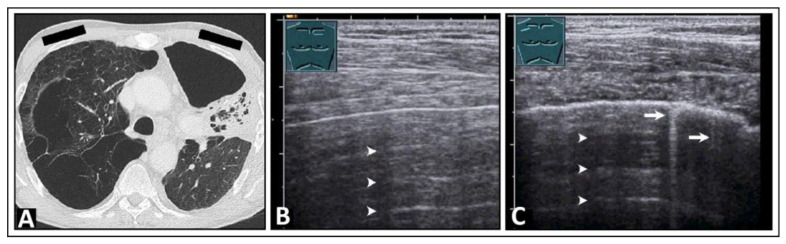
A patient with shortness of breath and known bullous emphysema in the context of chronic obstructive pulmonary disease following volume-reduction surgery due to recurrent pneumothoraxes. (**A**) On computed tomography, the bullous lung emphysema is shown with a large right apicoventral bulla; the black rectangles indicate the positions of the ultrasound probe. (**B**) On lung ultrasound, in the left upper lobe, a sharp standing reflective line with A-lines (arrowheads) without comet-tail artifacts is striking. A pneumothorax cannot be ruled out sonographically. (**C**) On lung ultrasound, the right upper field shows a somewhat rough pleura with comet-tail artifacts (arrows) and A-lines (arrowheads).

**Figure 5:**
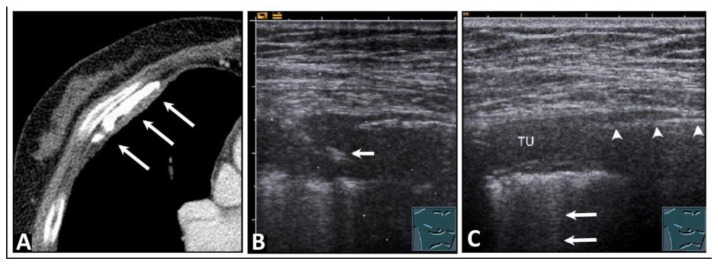
A patient who experienced occupational exposure as a cotton spinner. (**A**) Thickening of the pleura and calcifications are seen on computed tomography (arrows). (**B**) An ultrasound-guided histologic confirmation of the diagnosis of chronic pleurisy calcarea was performed. The arrow indicates the biopsy needle. (**C**) On lung ultrasound, there is pleural consolidation (TU) on the right side, multiple comet-tail artifacts posteriorly (arrows), and a distinct sharp echogenic line (arrowheads), ruling out a local pneumothorax, which was attributed to pleural calcification that caused complete dorsal sound extinction.

A pneumothorax can be ruled out if a lung-sliding, smooth, pleural reflection band and existing CTAs are seen. However, there are pitfalls of using LUS in the diagnosis of a pneumothorax, such as bullous lung emphysema (Figure 4), pleural calcification (Figure 5), or pseudo-B-lines in the case of a hydropneumothorax [20,21]. When dealing with a hydropneumothorax, the bubbles situated at the air–effusion interface near the parietal pleura can create a vertical reverberation artifact that resembles a B-line. Additionally, the movement of this interface between air and pleural effusion during the respiratory cycle of the patient might resemble lung sliding [21].

## 4. Pulmonary Edema and Acute Respiratory Distress Syndrome

A systematic review with meta-analysis of 1827 patients indicated that LUS had a sensitivity of 88% and a specificity of 90% for pulmonary edema, whereas chest radiography had a sensitivity of 73% and a specificity of 90% [3]. Pulmonary edema can be cardiogenic or non-cardiogenic, and its identification is a diagnostic puzzle. Clinically, patients have signs of heart failure, such as dyspnea and tachypnea pedal edema, anasarca, and cyanosis of the lips [22,23]. On ultrasound, patients show indirect signs of volume loading, such as a dilated inferior vena cava with lack of respiratory collapse and pleural effusions [24]. With the use of LUS, pulmonary edema can be diagnosed by direct signs. In pulmonary edema, the fluid first appears in the interstitium and then in the alveolar space [23]. Therefore, the auscultatory signs appear at a later stage of pulmonary edema. Ultrasound can overcome these limitations and detect pulmonary edema in its early stages.

**Figure 6:**
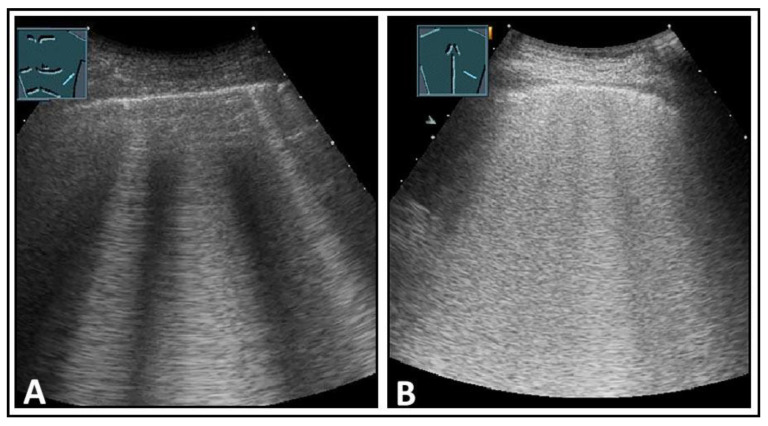
Depiction of pathologically increased B-lines. (**A**) Multiple B-lines; (**B**) multiple confluent B-lines in the “white lung” pattern.

**Figure 7:**
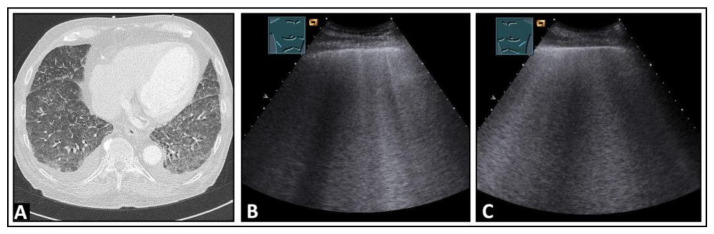
A patient with aortic valve insufficiency, progressive dyspnea, and cardiac pulmonary edema. (**A**) On chest computed tomography, bilateral pleural effusion and diffuse ground-glass opacities with septal thickening are visualized, consistent with congestion. (**B**,**C**) On lung ultrasound, increased B-lines are present bilaterally in the lower fields.

In the clinical picture of left heart failure with fluid overload and predominant appearance in the lower fields, the IP has also been termed the “sound of lung water”. This pattern can be represented either as multiple B-lines or as multiple confluent B-lines (Figure 6 and Figure 7) [25]. With confluent B-lines, the so-called “white lung” pattern is evident (Figure 6B).

**Figure 8:**
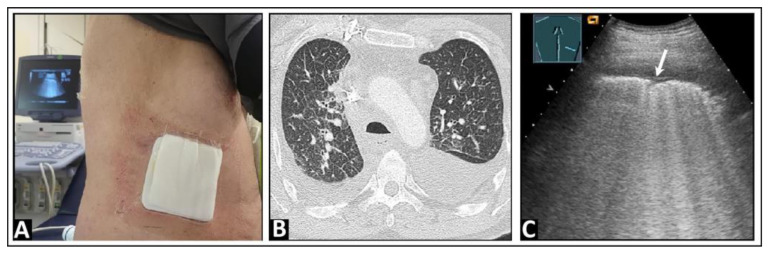
(**A**) A patient with an indwelling pleural catheter in place for 6 weeks due to a malignant effusion from a bronchial carcinoma and with exertion and speech dyspnea. (**B**) On chest computed tomography, multiple nodular consolidations are visualized, consistent with metastases, as well as septal thickening (right > left) indicative of lymphangitic carcinomatosis. (**C**) On lung ultrasound, the “white lung” pattern is observed, with a rough lung surface (arrow) and a delicate anterior pleural effusion.

**Figure 9:**
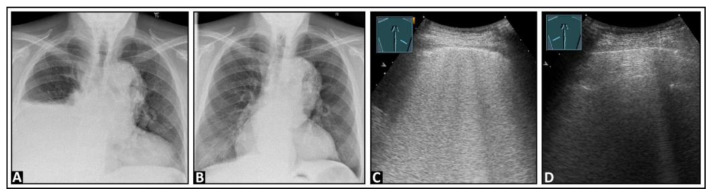
A patient with dyspnea and right-sided pleural effusion shown on chest X-ray images before (**A**) and after (**B**) an effusion-relieving puncture. (**C**) After puncture, on lung ultrasound, multiple confluent B-lines appear on the right side, suggesting a “white lung” pattern, as seen in re-expansion edema; (**D**) the left lung shows an inconspicuous pleural reflection band.

A pulmonary edema can also occur after a prolonged pleural drainage (Figure 8) or in the form of re-expansion edema following pleural effusion drainage (Figure 9). Re-expansion edema might be attributed to the dynamics between the heart and lungs during and following the process of draining fluid from the pleural cavity [26]. While draining, there is a reduction in both pleural and intrathoracic pressures, leading to an increase in left ventricle afterload, along with a corresponding decrease in the systolic performance of the left ventricle [26].

**Figure 10:**
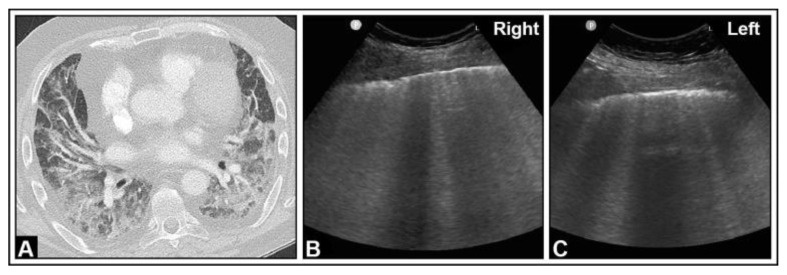
A patient with granulomatosis with polyangiitis undergoing rituximab and cyclophosphamide therapy, currently presenting with COVID-19 pneumonia and severe acute respiratory distress syndrome according to the Horovitz Index. (**A**) In the chest computed tomography, areas with lung consolidations and ground-glass opacities are present bilaterally. (**B**,**C**) In the B-mode ultrasound, multiple B-lines can be seen in both the right lung and the left lung.

Another cause of the appearance of an IP is acute respiratory distress syndrome (ARDS). ARDS results from protein-rich pulmonary edema leading to significant hypoxemia and impaired carbon dioxide elimination [27], and its diagnosis is made through clinical criteria in combination with chest imaging [28]. ARDS presents with an IP on LUS (Figure 10) [16], which has been shown to have a sensitivity of 93–98% and a specificity of 78–100% in the diagnosis of ARDS [29,30,31]. Another benefit of ultrasound is its use as a rapidly available and cost-effective method in the follow-up of ARDS [16].

## 5. Pneumonia

**Figure 11:**
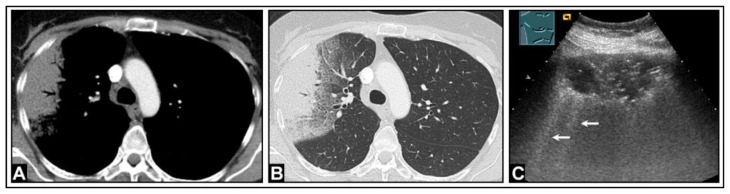
A patient with fever, auscultatory crackles, elevated inflammatory markers, and clinical suspicion of community-acquired pneumonia. (**A**,**B**) Computed tomography reveals consolidation with ground-glass opacities in the transitional area between consolidated and normal lung tissue. (**C**) Lung ultrasound demonstrates hypoechoic consolidation with air bronchogram; the border of the lesion to the lung appears blurred, with multiple partially confluent B-lines (arrows) originating from the interface.

Ultrasound has a high performance in the diagnosis of pneumonia. A systematic review with meta-analysis of 742 patients showed that LUS had a sensitivity of 95% and a specificity of 90%, whereas chest radiography had a sensitivity of 77% and a specificity of 91% [3]. Furthermore, a previous study demonstrated that LUS significantly enhances diagnostic performance in elderly patients with comorbidities [32]. The manifestation of pneumonia depends on the underlying pathogen [33]. A community-acquired lobar pneumonia presents on the LUS as consolidations with a positive air bronchogram (i.e., visibility of air-filled bronchi within areas of consolidation) [1,34,35]. In this context, an IP in the area of the transition from a visible consolidation to a normally ventilated lung may present as a transitional phase corresponding to ground-glass opacities on computed tomography (Figure 11).

**Figure 12:**
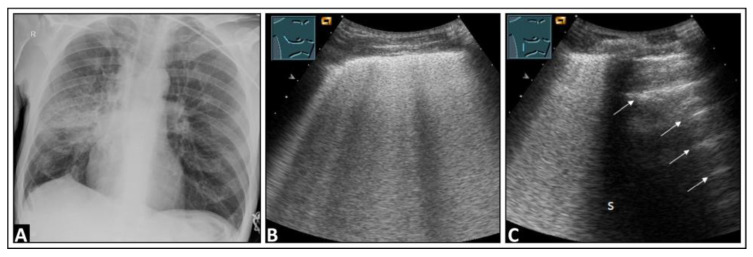
A patient with dyspnea, cough, elevated inflammatory markers, and right-sided pneumonia in the mid-zone observed on a chest X-ray (**A**). (**B**) On lung ultrasound, multiple confluent B-lines are visible, indicative of a “white lung” pattern. (**C**) There are a few intercostal spaces deeper than in panel B, there is an increase in B-lines cranially, and a normal lung is observed caudally with corresponding A-lines (arrows), similar to a localized interstitial pattern; dorsal sound extinction (s) is observed behind a rib.

**Figure 13:**
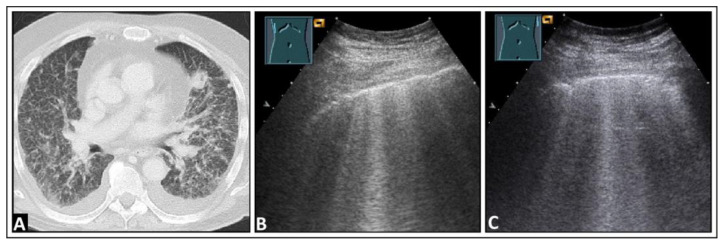
A patient experiencing dyspnea and elevated inflammatory markers, with a histologically confirmed lung infection indicative of nocardiosis following a history of bone marrow transplantation. (**A**) On computed tomography, disseminated small-spot consolidations in both lungs extend to the periphery with cavitary lesions. (**B**) On lung ultrasound, multiple B-lines are observed both on the right side and (**C**) on the left side, consistent with a diffuse interstitial pattern.

**Figure 14:**
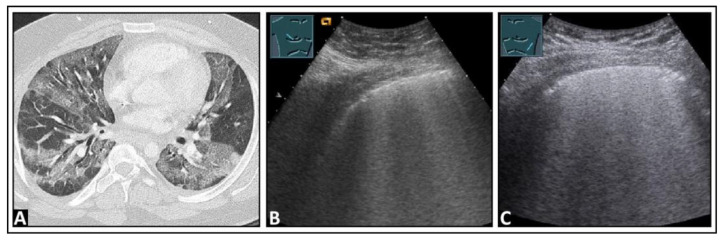
A patient with confirmed COVID-19 infection with progressive respiratory failure. (**A**) The computed tomography scan displays extensive bilateral ground-glass opacities, in some areas also emphasizing the interlobular septa (crazy paving), consistent with COVID-19 pneumonia. (**B**) On lung ultrasound, both on the right side and (**C**) on the left side, there are multiple confluent B-lines, indicating a “white lung” pattern consistent with a diffuse interstitial pattern.

**Figure 15:**
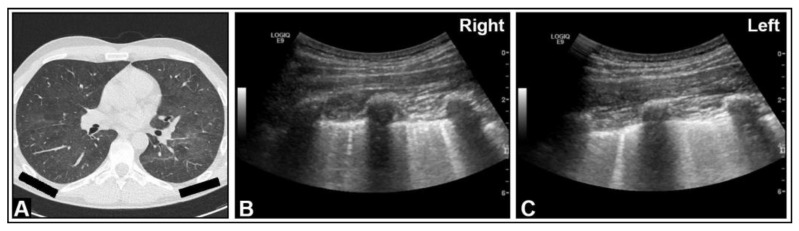
An HIV-positive patient with dyspnea and *Pneumocystis carinii* pneumonia. (**A**) On chest computed tomography, diffuse ground-glass opacities are observed. (**B**,**C**) On B-mode ultrasound, B-lines are visible in the right and left lungs.

The presence of an IP in the form of B-lines is a key characteristic in patients with atypical pneumonia [33]. An IP can be present in a bacterial pneumonia (Figure 12 and Figure 13), a viral pneumonia (Figure 14), or a fungal pneumonia (Figure 15).

## 6. Interstitial Lung Disease

**Figure 16:**
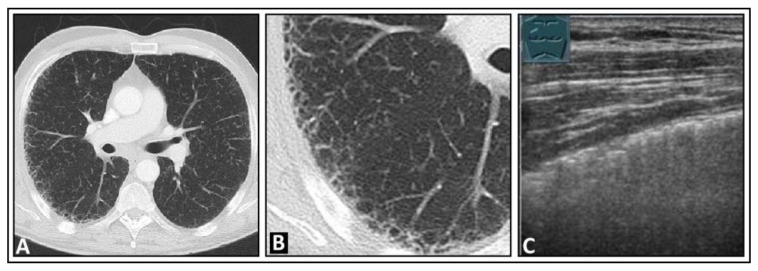
A patient with exertional dyspnea and confirmed idiopathic pulmonary fibrosis. (**A**) The chest computed tomography displays subpleural fine reticular changes in the upper lobe leading to honeycombing, with the right side (**B**) more affected than the left. (**C**) On lung ultrasound, multiple confluent B-lines are seen on the right side, with a rough lung surface.

In the diagnosis of interstitial lung diseases (ILDs), high-resolution computed tomography is considered to be the gold standard. Compared with computed tomography, ultrasound demonstrates high sensitivity but relatively low specificity [36]. Cogliati et al. reported a sensitivity of 92% and a specificity of 56% for LUS in detecting ILDs [37]. Therefore, the primary role of ultrasound is as a screening method. The sonographic image is characterized by an IP (Figure 16) due to chronic subpleural interstitial changes with reduced ventilation in the lung periphery [36].

## 7. Other Pathologic Situations

**Figure 17:**
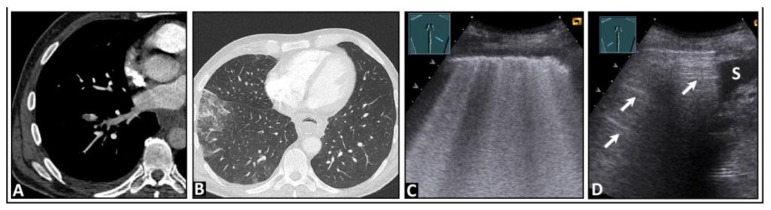
A patient experiencing right-sided, respiration-dependent thoracic pain due to a right-sided central pulmonary embolism. (**A**,**B**) The chest computed tomography scan shows bilateral pulmonary embolism, more pronounced on the right, especially affecting the lower lobe, with the onset of infarction pneumonia in the right lower lobe. (**C**) On lung ultrasound, there are multiple confluent B-lines on the right side, indicating a “white lung” pattern with a rough lung surface. (**D**) The left side displays a normal lung surface with A-lines (arrows); S = spleen.

**Figure 18:**
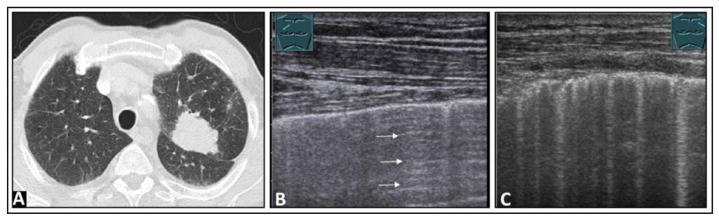
A patient with dyspnea on speaking, thoracic pain, and a confirmed left-sided intraparenchymal bronchial carcinoma located in the upper lobe, as seen on chest computed tomography (**A**). (**B**) On lung ultrasound, the right side displays an almost normal lung surface with A-lines (arrows). (**C**) Ventral to the centrally located lung tumor, given knowledge of the computed tomography findings, multiple vertical artifacts are evident in the form of B-lines and comet-tail artifacts, as seen in a focal interstitial pattern.

**Figure 19:**
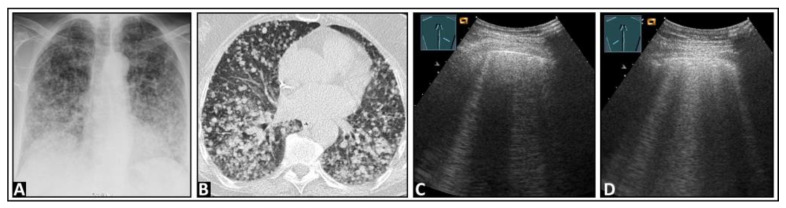
A patient with cough, exertional dyspnea, and histologically confirmed diffuse lung metastasis from a neuroendocrine tumor of the pancreas, showing on (**A**) chest X-ray and (**B**) chest computed tomography. (**C**) On lung ultrasound, multiple B-lines are observed, both (**C**) on the right side and (**D**) on the left side.

**Figure 20:**
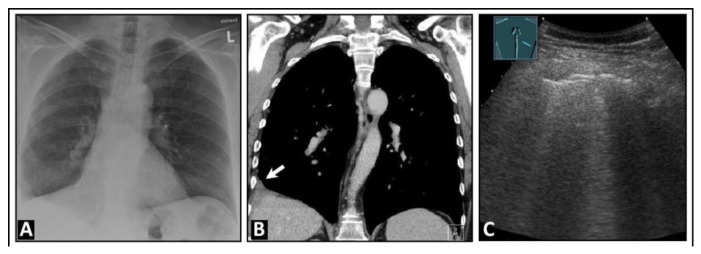
A patient experiencing pain in the right thoracic region and an obscured right diaphragmatic costal angle on (**A**) chest X-ray and (**B**) chest computed tomography (indicated by arrow). (**C**) On lung ultrasound, multiple B-lines (arrows) are seen in this area, accompanied by a rough, interrupted pleural reflex line (arrowheads), consistent with a focal interstitial pattern, most likely due to scarring.

**Figure 21:**
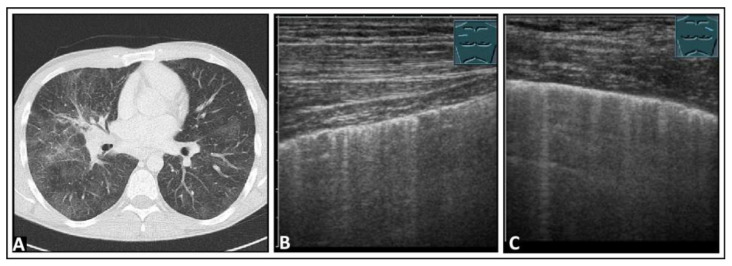
A patient with restrictive lung function test results and histologically confirmed pulmonary sarcoidosis. (**A**) The chest computed tomography reveals patchy ground-glass lung parenchyma changes with partly nodular thickening of the interlobular septa. (**B**) On lung ultrasound, both on the right side and (**C**) on the left side, multiple B-lines and comet-tail artifacts are observed, consistent with a diffuse interstitial pattern.

**Figure 22:**
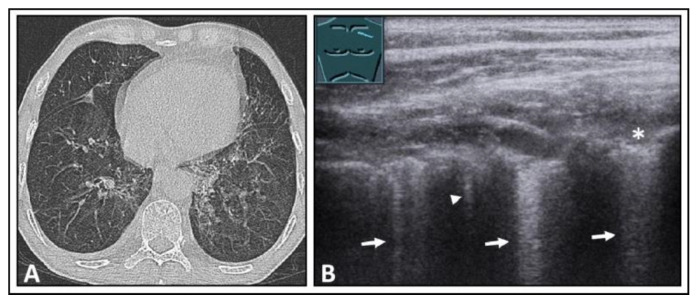
A patient with secondary acute myeloid leukemia following allogeneic bone marrow transplantation, with graft-versus-host disease (GVHD) of the intestines and increasing dyspnea. (**A**) The computed tomography scan shows mosaic-like ground-glass opacities on both sides, emphasized in the upper lobes, as well as peribronchial consolidations, consistent with acute GVHD of the lung. (**B**) On lung ultrasound, multiple B-lines (arrows) and a comet-tail artifact (arrowhead) are seen in the area of the upper lobe, with a rough, interrupted pleural reflection line (*) as seen with a focal interstitial pattern.

An IP can be observed in various pathologies, including, but not limited to, pulmonary embolism (Figure 17), lung cancer (Figure 18), lung metastases (Figure 19), pleural scarring (Figure 20), sarcoidosis (Figure 21), acute graft-versus-host disease (Figure 22) of the lung, and pulmonary contusion [2,14,18]. Depending on the clinical presentation, the IP appears either diffusely distributed across both lungs or as a localized IP. In these pathologies, the IP seen on LUS may correspond to ground-glass opacity in computed tomography [38].

## 8. Pleural Artifacts with Simultaneous Presence of Consolidation

**Figure 23:**
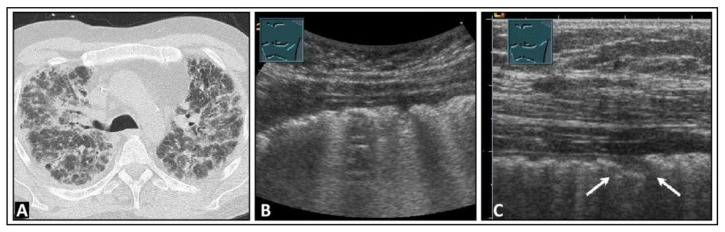
A patient with dyspnea and known advanced pulmonary fibrosis due to graft-versus-host disease following allogeneic stem cell transplantation. (**A**) The chest computed tomography scan displays severe fibrotic parenchymal changes. (**B**) On low-frequency lung ultrasound, multiple B-lines are observed on the left side, with a rough lung surface. (**C**) On high-frequency lung ultrasound, nodules up to 5 mm in size are observed (arrows).

**Figure 24:**
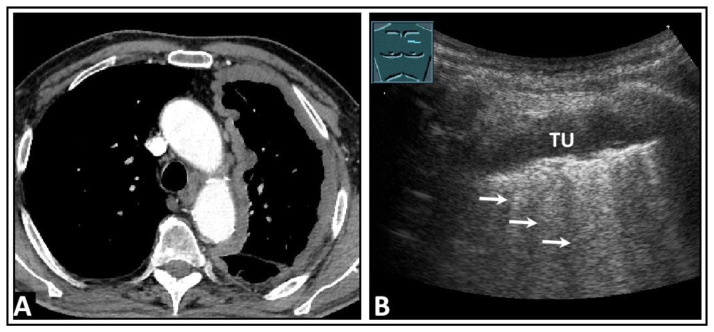
A patient with dyspnea, thoracic pain, and confirmed left-sided pleural mesothelioma on computed tomography (**A**). (**B**) On lung ultrasound, there is a widespread pleural consolidation (TU) on the left side, with multiple B-lines (arrows) and a rough lung surface adhering to the tumor layer.

**Figure 25:**
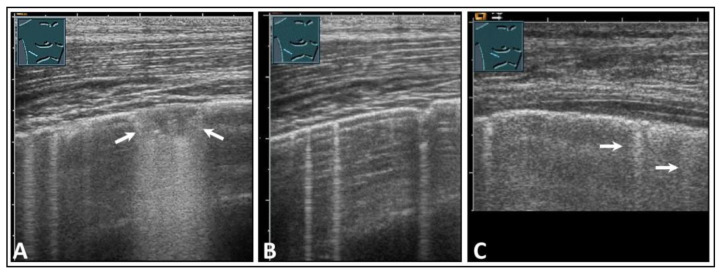
A patient with clinically diagnosed infectious pleuritis who presented with right-sided respiratory-dependent chest pain. (**A**) On lung ultrasound, a consolidation of approximately 5 mm with dorsal sound enhancement (arrows) was observed, along with (**B**) multiple B-lines (arrows), as well as (**C**) isolated comet-tail artifacts (arrows) with pleural thickening and a delicate effusion.

Pleuropulmonary pathologies with a decreasing air content must be differentiated from an IP, with <10% caused by alveolar consolidation of various etiologies [4]. In contrast to pleural roughness, solid pleural based focal lesions have a size of >5 mm [39]. In this context, the direct LUS visualization and characterization of pleural pathologies for diagnosis have come to the forefront. The detection of interface artifacts with adjacent lung tissue loses its diagnostic value here (Figure 23, Figure 24 and Figure 25).

## 9. Discussion

As this pictorial essay demonstrates, LUS has high sensitivity but lacks specificity in the detection of various interstitial lung pathologies based on the presence of IPs. Due to its high sensitivity, LUS can be used to rule out pulmonary edema, monitor the course of therapy in pulmonary edema, control fluid balance in shock patients, and for early detection of an ILD in patients with a corresponding clinical background, such as connective tissue diseases like systemic sclerosis, Sjögren’s syndrome, and antisynthetase syndrome or other rheumatoid diseases [2,40,41,42,43,44,45]. Although some studies have observed high diagnostic accuracy for LUS-related monitoring of ILDs, LUS appears to be unsuitable for ILD follow-up, given its lack of standardized scan views, inherent subjectivity, and limitation to examining only 70% of pleura-adjacent areas of the lung [46,47]. In diagnosing and screening for COVID-19 infection, LUS is deemed unsuitable due to its non-specificity. A prospective study has already demonstrated that the sensitivity of LUS varies depending on disease prevalence and patient demographics [48]. At present, there are limited data regarding the exclusion of ILD when an IP is absent and the role of IPs in the follow-up of patients after lung transplantation. However, initial data indicated a notable correlation between the B-line score and the identification of primary graft dysfunction in patients following lung transplantation. LUS was more effective than chest X-rays, suggesting that LUS could be a valuable tool for diagnosing primary graft dysfunction in post-lung-transplant patients [49]. Furthermore, at present, terms such as “B-lines” and “CTAs” are used heterogeneously and sometimes synonymously in the literature. Different interpretations of artifacts may lead to different therapeutic consequences. To date, there have been limited data regarding the validity of LUS in evaluating IPs while considering intra- and inter-observer variability.

When BLAs or CTAs are present, it is essential to consider clinical factors, such as the patient’s age and medical history, as well as device-dependent factors, such as transducer frequency and the presence of a device test. Only when these factors are considered can these artifacts be interpreted as a pathological IP. Fundamentally, the sensitivity and specificity of a diagnostic procedure depend on the clinical pre-test probability, the prevalence of the disease, and the patient spectrum [48]. In addition, LUS is an examiner- and device-dependent imaging modality, the benefit of which depends on interpretation by the physician [50]. In the event of ambiguity after clinical evaluation, LUS, and chest X-rays, further overview imaging, preferably a high-resolution chest computed tomography scan, is indispensable.

## 10. Limitations of LUS

In addition to the non-specificity in the interstitial pattern, the general limitations of LUS should be considered. In LUS, only approximately 70% of the pleural surface can be examined due to air and bony structures [51]. Furthermore, only peripheral pathologies in the lung can be examined. LUS is an operator-dependent method, and the results are influenced by the examiner’s experience and the device settings [3,16]. Moreover, the examination is patient-dependent. In overweight patients, examining the lungs can be challenging due to the thickening of the soft tissues [16].

## 11. Conclusions

Horizontal interface artifacts have limited clinical relevance. Increased vertical focal or diffuse interface artifacts in the form of an IP with CTAs or B-lines, or both, can frequently be detected with high sensitivity on LUS at the visceral pleura. These artifacts may have clinical significance. In most cases, additional radiological overview imaging is necessary for further evaluation. Due to the lack of specificity of LUS, clinical classification of the artifacts is essential, and the interpretation is possible and meaningful only depending on the clinical pre-test probability. Furthermore, to categorize the pathologies associated with these patterns and to investigate the significance of these pathologies in standardized studies, it is imperative that the nomenclature is consistently defined and applied.

## Data Availability

Not applicable.
